# Gene expression in male and female stickleback from populations with convergent and divergent throat coloration

**DOI:** 10.1002/ece3.8860

**Published:** 2022-04-29

**Authors:** Jeffrey S. McKinnon, William Burns Newsome, Christopher N. Balakrishnan

**Affiliations:** ^1^ Department of Biology East Carolina University Greenville North Carolina USA

**Keywords:** carotenoid, gene expression, sexual dimorphism, stickleback

## Abstract

Understanding of genetic mechanisms underlying variation in sexual dichromatism remains limited, especially for carotenoid‐based colors. We addressed this knowledge gap in a gene expression study with threespine stickleback. We compared male and female throat tissues across five populations, including two in which female red coloration has evolved convergently. We found that the expression of individual genes, gene ontologies, and coexpression networks associated with red female color within a population differed between California and British Columbia populations, suggesting differences in underlying mechanisms. Comparing females from each of these populations to females from populations dominated by dull females, we again found extensive expression differences. For each population, genes and networks associated with female red color showed the same patterns for males only inconsistently. The functional roles of genes showing correlated expression with female color are unclear within populations, whereas genes highlighted through inter‐population comparisons include some previously suggested to function in carotenoid pathways. Among these, the most consistent patterns involved *TTC39B* (Tetratricopeptide Repeat Domain 39B), which is within a known red coloration QTL in stickleback and implicated in red coloration in other taxa.

## Graphical abstract

Understanding of genetic mechanisms mediating variation in sexual dichromatism remains limited, especially for carotenoid‐based colors. Comparing gene expression in skin tissue from five populations of stickleback, including two in which female red coloration has evolved convergently, we found extensive inter‐population differences in the genes showing red‐correlated expression in females. Nevertheless, TTC39B, which has been suggested to mediate carotenoid‐based coloration in several taxa and is located within a stickleback pigmentation QTL, was consistently associated with red throat color.

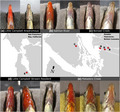

## 1 INTRODUCTION

Conspicuous ornaments are often sexually dimorphic, with greater exaggeration in males, but both ornament exaggeration and sexual dimorphism may vary greatly between even closely related species and populations (Andersson, 1994; Darwin, 1871; Lande, 1980). Variation in dimorphism may result from differences in the ornamentation of females, males, or both sexes (Badyaev & Hill, 2003). Elucidating the processes mediating ornament evolution in each sex, and the genetic mechanisms operating within and between the sexes, is therefore important to understanding the evolution of such traits. However, selection pressures and genetic mechanisms have been studied for ornaments of both sexes in few species, with female ornaments generally less studied than those of males—although the literature on female ornament evolution has expanded in recent years (Hare & Simmons, 2019; Kraaijeveld, 2014).

There are two major classes of explanation for conspicuous female ornaments. The first is that ornaments are advantageous to females, through one or more of inter‐sexual, intra‐sexual, or social selection (Hernández et al., 2021). The second, which was essentially the default explanation for decades and is sometimes known as the genetic correlation or correlated response hypothesis, is that female ornaments evolve as a response to selection on males, owing to shared genetic underpinnings (Hare & Simmons, 2019; Kraaijeveld, 2014; Lande, 1980).

Color patterns, the focus of the present work, are popular targets for studying the evolution of both ornaments and sexual dimorphism, as well as for the investigation of diverse aspects of natural selection (Kettlewell, 1955; Olendorf et al., 2006; reviewed in Cuthill et al., 2017; Orteau & Jiggins, 2020). The evolutionary genetics of color patterns were investigated extensively in the 20th and early 21st centuries using mendelian and quantitative genetic approaches (e.g., studies reviewed in McKinnon & Pierotti, 2010). With the spread of genomic methods, progress has accelerated in elucidating how specific color genes and pathways, some sexually dimorphic, evolve in a variety of systems. Carotenoid‐based ornaments are of particular interest because they have been the subjects of widely cited studies of sexual selection (Hill, 1991, Milinski & Bakker, 1990) and the focus of considerable theorizing, especially with regard to honest signaling and good genes hypotheses (e.g., Koch & Hill, 2018). Nevertheless, understanding of carotenoid evolutionary genetics remains limited and this is an area of active research (Orteau & Jiggins, 2020; Toews et al., 2017).

The subject of the present work, the threespine stickleback, is an important evolutionary model (McKinnon et al., 2019; McKinnon & Rundle, 2002; Reid et al., 2021). Stickleback color pattern evolution has been investigated with regard to the closely related topics of sexual selection (e.g., Milinski & Bakker, 1990; Tinghitella et al., 2018; Wright et al., 2015), parental care (Candolin & Tukiainen, 2015), and speciation (e.g., Blouw & Hagen, 1990; Boughman, 2001; Marques et al., 2017), as well as crypsis (e.g., Greenwood et al., 2012; Gygax et al., 2018). In particular, the carotenoid‐based anterior body coloration of male stickleback has been widely cited as a potentially honest signal of genetic quality (Milinski & Bakker, 1990). Male color has also been shown to vary with the light environment (Boughman, 2001; Brock et al., 2017; Reimchen, 1989) and to be targeted by some fish predators (Johnson & Candolin, 2017).

Orange‐red throat coloration has now been reported for female stickleback, as well as males, from at least three localities. These include widely separated drainages in which female color likely evolved independently from an ancestral state, in anadromous and/or marine populations, in which only males are red (McKinnon et al., 2000; Von Hippel, 1999; Yong et al., 2013, 2018). The genetics of female red throat color are of particular interest because there is so far no evidence that females with red throats are favored by sexual or social selection (Wright et al., 2015; Yong et al., 2013, 2015, 2018).

The genetic bases for convergent adaptations in sticklebacks have often been shown to result from reuse of the same genes and sometimes the same alleles (Bassham & Catchen, 2018; Jones et al., 2012; Kitano et al., 2019; Roberts‐Kingman et al., 2021; but see Fang et al., 2020), but whether convergent female ornaments result from the same or different genetic mechanisms is little studied in stickleback or in any other taxa (but see Yassin et al., 2016). While it is commonly assumed that genes and developmental mechanisms underlying similar traits in males and females are the same, this need not be the case. Indeed, van der Bijl and Mank (2021) report numerous examples from studies of mouse gene knockouts of cryptic sex differences in genetic architecture. Surprisingly, concordant regulatory changes may lead to discordant genetic effects in sexually monomorphic as well as dimorphic traits. In humans, genes with similar expression patterns in males and females may be regulated by different transcription factors and networks in each sex (Lopes‐Ramos et al., 2020).

In QTL analyses of a California stickleback population with red‐throated females, up to three genome regions mediated variation in female red throat coloration. The regions associated with male color were closely collocated and possibly the same. In addition, one region almost entirely overlapped a QTL for spine color, which is present in both sexes. The observation of extensively overlapping QTL for males and females is consistent with the hypothesis that female red throats arise largely as a correlated response to selection on males (Yong et al., 2016). Studies with other populations have found additional regions of the genome to be associated with variation in male color (Malek et al., 2012) and suggested a single‐locus genetic architecture for red versus black male nuptial coloration (Hagen & Moodie, 1979). Some melanin‐related genes have also been identified and characterized (Hart & Miller, 2017; Miller et al., 2007) in stickleback and the molecular genetic basis of variation in cryptic striping has been elucidated (Greenwood et al., 2011, 2012). In addition, correlations (in some cases genetic) have been documented between male red color and other traits including female mating preference (Bakker, 1993), female body condition (Bakker et al., 1999), aggressive behavior (Bakker, 1994; Wright et al., 2016) and male vision (Brock et al., 2018).

Here we report the results of a study of gene expression in the skin of male and female threespine stickleback from populations in which males, both sexes, or neither sex may possess carotenoid‐based throat coloration. Four populations are stream‐ and freshwater‐resident, three from British Columbia and one from California. A fifth population, from British Columbia, possesses an anadromous life history. Red female throat coloration has almost certainly evolved independently and convergently in the California and British Columbia stream‐resident populations (Yong et al., 2013, 2018). We address three main research questions. First, for British Columbia and California populations, is expression of the same or different genes and networks associated with convergent female throat color? Second, is female red throat coloration associated with expression of the same genes and networks as male red throat coloration, as predicted by the genetic correlation hypothesis? Third, for which genes is expression most strongly correlated with color, and are these genes known or candidate pigmentation genes, and/or associated with previously identified color QTL?

## 2 METHODS

### 2.1 Overview of analyses and contrasts

In our main analyses, we used DESeq2 (Anders & Huber, 2010) to: identify genes whose expression was correlated with female red coloration within populations; test whether the expression of the same genes was correlated with female color in different populations; ask if the genes showing expression correlated with female color also showed differential expression in analyses including both males and females. Because some red‐associated genes may differ in expression mainly between populations, we conducted complementary analyses comparing red females with dull females of different populations in which dull females predominate. To address similar questions at a systems level, we used WGCNA to ask if gene networks showing color‐correlated differential expression, in each population possessing red females, were present in the other population; we conducted an analogous analysis of the two sexes. In order to identify candidate genes for further study, we asked if any of the genes consistently associated with red coloration had been linked with coloration in other species, and if any were associated with stickleback coloration QTL detected in one of the same populations (Yong et al., 2016). As a first step before the main analyses, we characterized the overall structure of the data and broad patterns of variation, by population and sex, with a principal component analysis of gene expression.

### 2.2 Study populations

**FIGURE 1:**
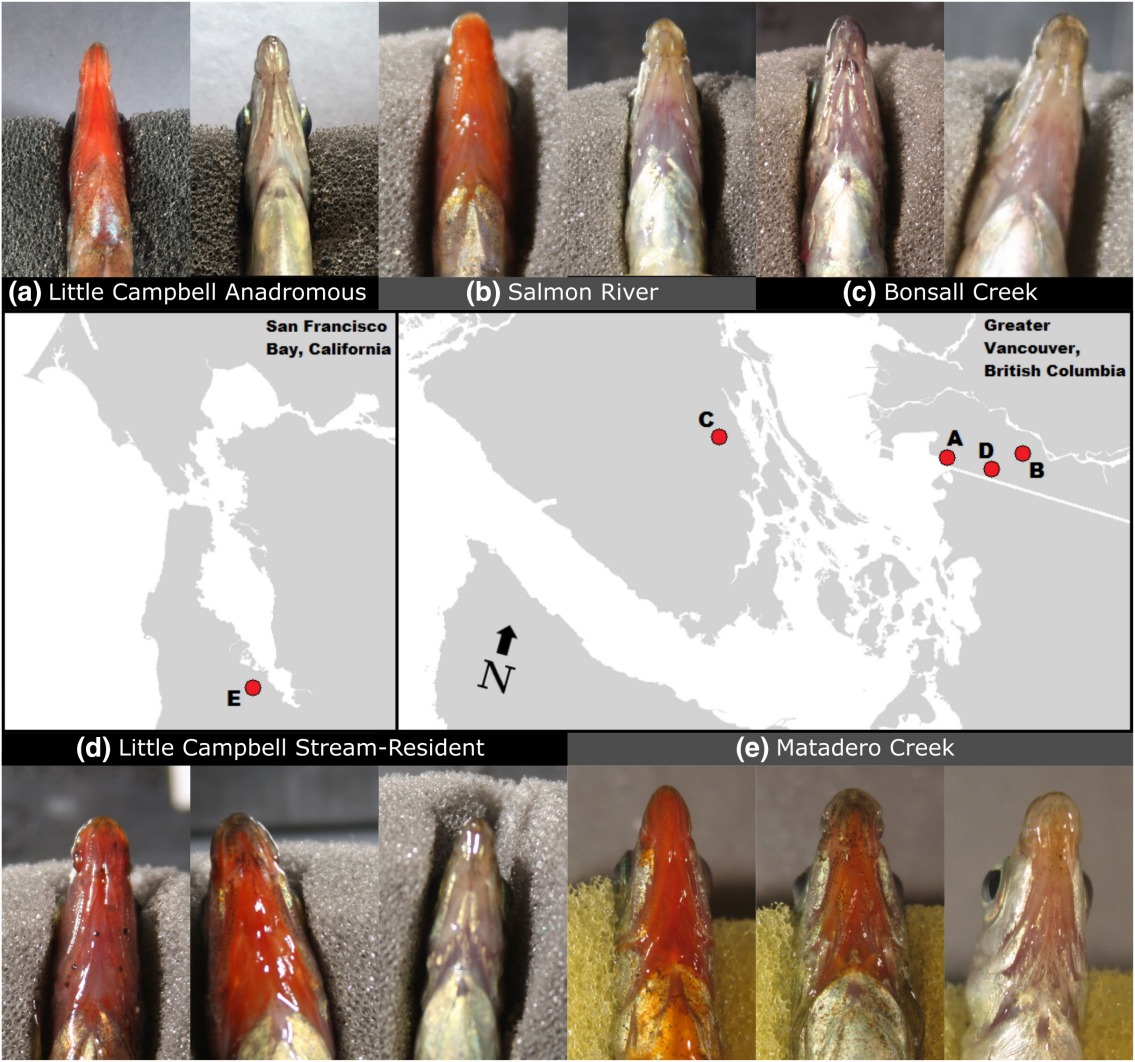
Map showing study populations and images of males and females of each population. Sites, images from left to right [sample size]: (a) Anadromous male [4], female [4]; (b) Salmon River male [5], female [4]; (c) Bonsall Creek male [4], female [4]; (d) Little Campbell Stream male [4, but no reflectance data for one], red female [5], dull female [3]; (e) Matadero Creek male [6], red female [4], dull female [4]

Fish were collected from populations in British Columbia, Canada, and California, USA (Figure 1). Populations surveyed are Little Campbell Anadromous (49°00'58 N, 122°46'46 W; henceforth “Anadromous”), Little Campbell Stream‐resident (49°00'43 N, 122°37'30 W), Salmon River (49°05'29 N, 122°29'34 W), Bonsall Creek drainage (48°51'05 N, 123°42'40 W), and Matadero Creek (the only California population: 37°23'36 N, 122°9'46 W). Using minnow traps and seine/dip nets, fish were collected from the field in spring 2014 and transported to East Carolina University, North Carolina. British Columbia stickleback were collected in late April and California stickleback were collected in June/July. Fish from Little Campbell Stream‐resident (henceforth LC Stream) and Matadero populations, both possessing red females, were sampled so as to ensure high diversity in female throat coloration, with sufficient numbers of both red and dull females (which were categorized as red or dull for some analyses). All fish were held for two weeks to allow acclimation to the laboratory. They were kept in 102‐liter tanks at approximately 15–20 fish per tank under natural‐spectrum mimicking fluorescent light (Lumichrome^®^ Full Spectrum Plus, Lumiram Electric Co., Larchmont, NY, USA) and photoperiod at 17–20°C. Fish were fed bloodworms (chironomid larvae) and brine shrimp twice per day.

### 2.3 RNA extraction, library preparation, sequencing, and generation of read counts

Red coloration was assessed following protocols described in Yong et al. (2013) using an Ocean Optics Maya2000 Pro spectrophotometer. Prior to collection of spectrometry data, subject fish were euthanized by MS‐222 overdose. Immediately following these measurements, throat and telencephalon tissue (these results to be presented in a different MS) were removed and stored in RNAlater. “Throat tissue” specifically refers to ventral surface tissue between the opercula and immediately ventral to the ceratohyal cartilage, excluding tissue immediately ventral to the sternohyoideus; this includes (potentially) red‐colored skin tissue on the underside of the head, detached from underlying cartilage. Additionally, standard length was measured and each fish was dissected to confirm sex. The remaining tissue samples were immersed in RNAlater and stored in a −80°C freezer for later use. Animal use protocols were approved by East Carolina University's Institutional Animal Care & Use Committee (AUP #D224b) and experiments were performed in accordance with relevant institutional and national guidelines for the care and use of laboratory animals.

Tissues were homogenized in TRI reagent with a Bio‐Gen 2000 Homogenizer (Pro Scientific) and RNA was extracted using the TRI Reagent protocol. Samples were then treated with DNase to remove contaminant DNA, and total RNA isolation was purified using the manufacturer's instructions for the RNeasy Kit (Qiagen). To determine RNA quality, RNA samples were run on a RNA 6000 picoLab Chip using a 2100 Bioanalyzer (Agilent). The average RNA quality for the samples used in this study was high (RNA integrity number, RIN, = 8.1). RNA was extracted from throat and brain tissue and stored in a −80°C freezer until sequencing. cDNA library preparations were conducted by the University of Illinois Roy J. Carver Biotech Center under the guidance of Dr. Alvaro Hernandez using Illumina TruSeq SBS sequencing kit version 4. Ninety‐six libraries (52 throat, 44 telencephalon) were multiplexed on a full flow cell (8 lanes) Illumina HiSeq 2500 to yield approximately 18.8 million single‐end, 100bp reads per library. Fastq files were generated and demultiplexed with the bcl2fastq v1.8.4 Conversion Software (Illumina).

Following sequencing, standard quality control (adapter removal and trimming of low‐quality bases) was conducted using TrimGalore on default settings (Krueger, 2015; Martin, 2011). Reads were mapped to the stickleback reference genome (Jones et al., 2012) using Tophat version 2.0.13 (Trapnell et al., 2009). Tables of read counts, which were used as input for most downstream analyses, were generated using HT‐Seq version 0.6.0 (Anders et al., 2015). They referenced Ensembl 79. Gene annotations were from Ensembl 100 unless otherwise indicated.

### 2.4 Color measurement and analysis

Reflectance spectra were processed with *pavo* (Maia et al., 2013) in the R environment (R Core Team, 2017) using a perceptual model of threespine stickleback vision (Stuckert, Drury, et al., 2019) to generate a measure of chroma, r.vec, which can be thought of as color intensity, and two indices of wavelength, h.phi and h.theta. The r.vec variable, which in our analyses is a measure of red intensity and will henceforth be referred to as chroma, was the intended focus but all three variables proved to be strongly correlated (see *Results*). Some heteroscedasticity was present in chroma data owing largely to limited variance in the consistently low values of the Bonsall population (note that males in this site appear to be melanic based on our field observations; we have observed some red expression in males from this drainage held in the laboratory for an extended period, but not in males in the present study). This was not completely eliminated by transformations and significance values were high and robust, so analyses of raw data, conducted using JMP Pro 14.1.0, are presented for ease of interpretation. Reflectance data were not available for one Little Campbell Stream male, but that male's RNAseq results were retained in most analyses as other key data were present.

### 2.5 Differential expression analyses

To characterize major patterns of variation in expression data, principal component analyses (PCA) were run using the plotPCA function of DE‐Seq2 version 1.24.0 (Anders & Huber, 2010). We then tested for effects of population and sex using ANOVA, in JMP Pro 14.1.0. ANOVA results for PC1 were checked and confirmed with non‐parametric Wilcoxon tests as some heteroscedasticity was present and not eliminated by standard transformations. ANOVA results are reported for consistency.

Differential expression analyses were conducted using DE‐Seq2 version 1.24.0 (unless otherwise indicated; Anders & Huber, 2010), which tests for differential gene expression using a negative binomial general linearized model. Effects in DE‐Seq2 are presented as log_2_ fold change, which for an experiment is log_2_ (treated/untreated); for continuous independent variables, as in some of our analyses, the reported log_2_ fold change is per unit of change in the continuous variable. Unless otherwise indicated, alpha values for DESeq2 analyses have been corrected for multiple testing using the method of Benjamini and Hochberg (1995) and are denoted as “padj.” We accepted the DESeq2 default independent filtering of lowly expressed genes, which optimizes the number of adjusted p‐values, resulting in a padj of “NA” for some genes. Uncorrected significance tests are included in supplemental tables as a complement to corrected tests. Provisional characterizations of genes not named in Ensembl are presented when highly significant or otherwise of interest. Because some populations do not have red females and only one population lacks red males, our sample design is not balanced. We therefore conducted our analyses through focused comparisons rather than through a comprehensive model.

#### 2.5.1 Analysis of red females in Matadero and LC Stream populations

We first tested whether expression of the same or different genes was associated with convergent female red throat coloration within LC Stream (BC) and Matadero (California) females. Red chroma was treated as a continuous variable to maximize power. (A) We asked if there were significant interactions between red chroma and population/region in gene expression. Genes that show a significant interaction have distinctive associations with color among populations, as predicted if the genes differ. If genes are the same, only significant main effects are expected. (B) We tested for associations between gene expression and chroma within each of the two populations, after significant interactions between chroma and population proved common.

#### 2.5.2 Comparisons of red females and red males

Next, we tested whether genes whose expression correlated with red in females (1B above) were also associated with red across the sexes, as predicted by the genetic correlation hypothesis. Correlations between chroma and gene expression were analyzed for males and females together, first with a univariate analysis and then with sex included as a covariate, and the results compared with those for genes identified in 1B. The analysis was then repeated with the addition of an interaction term for sex*red chroma, to test for different relationships between chroma and gene expression in males versus females. These within‐population analyses were conducted using DESeq2 1.32.0. In addition, we assessed whether in inter‐population contrasts, males and red females of the same populations differed in expression of the same genes when contrasted with females from populations lacking red in females (as in 3 below).

#### 2.5.3 Gene expression comparisons of red females with dull females from other populations

Because genes mediating variation in a trait within and between populations are not necessarily the same, we also conducted an “inter‐population” analysis comparing red females (dull females were excluded to ensure a meaningful contrast) from each of LC Stream and Matadero against females from each of two freshwater‐resident populations in which red females are less common (Salmon River) or absent (Bonsall Creek). That is, we compared red females from LC Stream with dull females from Salmon and with those from Bonsall, and the same for Matadero females. While these contrasts are expected to reveal genes that differ by population independent of color, genes associated with color will also be encompassed, especially genes that differ in expression between red females and both of the dull female populations.

### 2.6 Functional enrichment testing

Sets of genes differentially expressed with red coloration in females of each red female population were tested for functional over‐representation using GOrilla (November 25–30, 2020), which tests rank‐ordered gene lists for functional enrichment toward the top of the list (Eden et al., 2007, 2009). To run these analyses, stickleback Ensembl gene stable IDs were converted to those for zebrafish, which for some genes resulted in longer lists owing to zebrafish possessing multiple members of a gene family that all correspond to a single stickleback gene stable ID. To avoid artifacts we retained only the first (ordered by name) in the list of zebrafish genes corresponding to a stickleback gene; these results are presented. We tested the robustness of the results obtained by instead retaining only the last gene in the zebra fish list and rerunning the analysis. Using default GOrilla settings, padj of 0.05, and the “Process” ontology, the results were identical for the number of Gene Ontology terms shared between analyses with Matadero and with LC Stream females.

### 2.7 Candidate gene annotation and analysis

Because some pigmentation pathways may not be thoroughly covered by gene ontology analyses (Baxter et al., 2019), we also compared differentially expressed genes with a list of candidate pigmentation genes associated with pigmentation/color phenotypes. First, we compiled stickleback genes orthologous to 650 genes in a manually curated list associated with integumentary pigmentation phenotypes in humans, mice, and zebrafish (Baxter et al., 2019). We used Ensembl (98; Sep. 29, 2019) to search for stickleback orthologs, retaining those which Ensemble assigned high orthology confidence (1 rather than 0). Of the 619 zebrafish genes in the list, we retained 526 stickleback orthologs; of 30 mouse genes lacking zebrafish homologs, we retained four; one human gene with neither a zebrafish nor mouse homologue lacked a stickleback homologue.

As a supplement to this extensively curated list of 530 genes, we used stickleback orthologs of genes in a color gene list assembled by Stuckert, Moore, et al. (2019), derived from a broader array of taxa but less extensively annotated and vetted. We first used Ensembl to search for stickleback orthologs corresponding to gene names. To be thorough, we then used the list to search for human and zebrafish genes, in turn using those to search for stickleback orthologs. We retained those with a stickleback gene name which Ensembl assigned high orthology confidence (1 rather than 0); we merged the three lists and removed duplicate genes. We augmented this set with genes associated with carotenoid pigmentation based on Toews et al. (2017), again using Ensembl to identify stickleback genes, a total of 10, corresponding to carotenoid‐related genes BCO1, BCO2, and CYP2J19 (though the latter is not expected to have direct homologues in fish); we used multiple stickleback genes from the same subfamilies where one to one orthology could not readily be established. *TTC39B* has been found to be pigment related (Ahi, Lecaudey, Ziegelbecker, Steiner, Glabonjat, et al., 2020; Hooper et al., 2019; Salis et al., 2019) so it was also added (and its second copy). The Stuckert, Moore, et al. (2019) and Toews et al. (2017) lists added 147 new genes. With Ensembl 100, some gene identifications changed such that 13 were removed, for a final total of 666 pigment‐related genes (final list in Table S1). We describe genes from this list as candidate pigmentation genes. In addition, genes were assigned to QTL Bayesian credible intervals from Yong et al. (2016) using the assembly update of Glazer et al. (2015).

### 2.8 WGCNA analyses

We conducted network analyses of RNAseq data using the R package WGCNA 1.68. Weighted gene correlation network analysis (WGCNA) is a systems biology method that can be used to find clusters, called modules, of genes with highly correlated expression (Langfelder & Horvath, 2008; Langfelder et al., 2011). We analyzed networks based on expression data transformed using DESeq2’s variance‐stabilizing transformation (Anders & Huber, 2010), with all genes not expressed in at least 50% of samples removed and a minimum module size of 30. We tested the correlation of modules with red intensity in R, using the method of Benjamini and Hochberg (1995) to correct for multiple tests.

As with DE‐Seq analyses, we used WGCNA to test for similarities and differences between Matadero and LC Stream populations. To do this, we built separate networks for each population, including males and females. Due to small sample sizes for LC Stream, we included the adjacent (and similar in overall gene expression) Salmon River population for network construction. The resulting modules were tested for associations with color (in both sexes and in females only). All modules, including color‐associated modules, were then tested for preservation of network structure among populations. It is important to keep in mind that both males and females were included in these WGCNA analyses, when interpreting relationships between WGCNA modules and traits in a single sex, and that sample sizes were relatively small for this method.

We also tested for network preservation between sexes to identify whether genes associated with color in females were preserved in males. To do this required larger sample sizes, so we built another gene co‐expression network for all of the British Columbia fish together. Using these modules, we tested for module preservation of color‐associated modules between males and females.

## 3 RESULTS

### 3.1 Variation in red coloration

**FIGURE 2:**
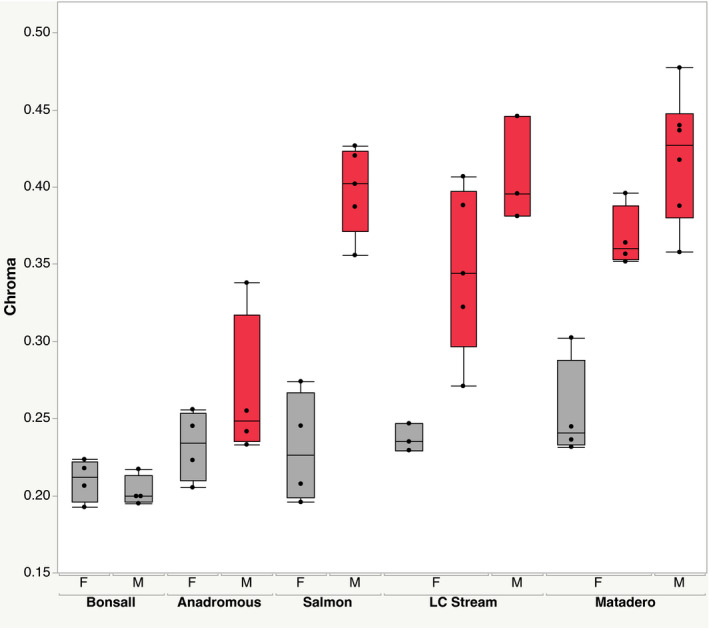
Red chroma for populations, sexes, and color groupings in this study. Fish classified *a priori* as red in red, dull in gray. Grouped by population (LC Stream, Little Campbell Stream‐resident; Matadero from California, others British Columbia), sex (M, male; F, female), and color pattern. Sample size = 50, with 3–6 fish in each sub‐category, shown as points in boxplots; boxplots show median, quartiles, ranges

Analyses of throat color using perceptual models confirmed that red chroma (the *Pavo* color measure r.vec) varied significantly between sexes and populations overall (two‐way ANOVA, sex: *F* = 32.6, df = 1, 40, *p* < .0001; population: *F* = 19.0, df = 4, 40, *p* <.0001; Figure 2), with a significant interaction between sex and population (*F* = 4.3, df = 4, 40, *p* = .0053). Both Bonsall sexes showed weak red intensity, as expected. Anadromous males showed intermediate color intensity, possibly owing to male coloration in that population varying more with nesting and social context (i.e. being especially weak without a nest: Rush et al., 2003) and males lacking opportunity to build nests. Within each of British Columbia's LC Stream and California's Matadero populations, red‐classified females were significantly more intensely red than dull females (LC Stream: *p* = .015, df = 6; Matadero: *p* = .0011, df = 6; Figure 2). Measures of hue were highly correlated with each other and with red chroma (in all cases *r* > 0.94 or less than −0.90, *p* < .0001, *n* = 50).

### 3.2 Major axes of variation in gene expression

RNA sequencing yielded a mean of 19.1 million reads per throat sample. On average ~83% mapped to the genome, and about 36% of mapped reads could be assigned to annotated genes. Reads that were not counted either mapped to multiple places in the genome, mapped outside of known features (*i*.*e*., genes), or could not be unambiguously assigned to genes.

**FIGURE 3:**
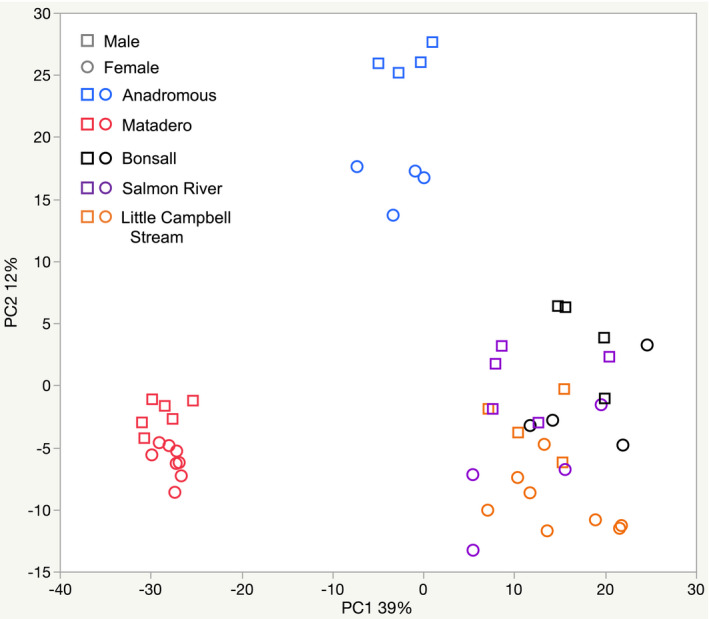
DESeq2 PC1 vs. PC2 for all populations. Males: square symbols; females: round symbols; red: Matadero stream (California; all other populations from British Columbia), includes red‐throated females; blue: Anadromous (only males red‐throated); black: Bonsall stream (all lack red throats); orange: Little Campbell Stream‐resident (includes red‐throated females), purple: Salmon River (only males red‐throated)

In a PCA of throat expression profiles, PC1 and PC2 explained 39% and 12% of the variance, respectively (Figure 3). Two‐way ANOVAs testing sex, population, and their interaction on PC1 and PC2 show significant effects of population on both PCs (PC1: *F* = 228.82, df = 4, 41, *p* < .0001; PC2: *F* = 168.22, df = 4, 41, *p* < .0001) and of sex on PC2 (*F* = 81.76, df = 1, 41, *p* < .0001; Figure 3) but not PC1 (*F* = 0.2746, df = 1, 41, *p* = .6031); the interaction term was not significant for either PC (PC1: *F* = 0.2560; PC2: *F* = 2.0030, df = 4, 41, *p* > .1 for both). For both PCs, the anadromous and freshwater populations are distinct and for PC1 the British Columbia freshwater populations were also well separated from the Matadero population. Males had higher mean values of PC2 across all populations, including the non‐red Bonsall population, though the pattern was especially strong for anadromous stickleback.

### 3.3 Are the same genes associated with red chroma for females of the Little Campbell Stream‐resident and Matadero populations?

For females of the two populations possessing red‐throated females, Matadero and LC Stream, we first tested for general relationships between red chroma and gene expression in a DESeq2 analysis with terms for red chroma and population. Red chroma was significant (*p*adj < .05) for only a single gene, ENSGACG00000019727, which was not in a QTL and was not assigned a name in Ensembl. However, when a term for the interaction between population and red chroma was added, it was significant for the expression of 100 genes (Table S2). Separate analyses of females of each population also indicate that correlations between gene expression and chroma are notably different between females of the two populations, with the expression of 28 genes significantly correlated with chroma for females of the Matadero population and 83 for LC Stream females (*p*adj < .05), but no individual genes exhibiting significant expression‐correlations with chroma in both populations (Table S3).

Four of the genes correlated with red chroma in LC Stream females were from within the credible intervals of the QTL, and five for Matadero females, but with different individual genes for each population. Only one pigment‐associated gene was significant, *mcm6* in Matadero (associated with orange coloration in an Australian lizard, *Ctenophorus decresii*: McLean et al., 2017; Table S3).

A gene ontology analysis also supports the distinctiveness of color‐associated genes in each of LC Stream and Matadero populations (Tables S4 and S5). For example, the top three (Process) terms for LC Stream were proteasomal ubiquitin‐independent protein catabolic process; macromolecule catabolic process; cellular macromolecule catabolic process. In contrast, the top three terms for Matadero were rRNA metabolic process; rRNA processing; nucleic acid metabolic process. No terms directly associated with pigmentation were significant.

WGCNA analysis of LC Stream fish resulted in 28 co‐expressed gene modules. The module eigengene, the first principal component, showed a significant correlation with red chroma for three (Table S6). No modules correlated significantly with red chroma for just females (Table S7). When LC Stream modules were tested for preservation against the Matadero data set, Zsummary values ranged from 0.24 to 31.2, with three under two and therefore not preserved, 12 between two and 10 and therefore preserved but not strongly, and 13 over 10 and thus strongly preserved (Langfelder et al., 2011; Table S8). All modules exhibited high “quality,” a measure of internal preservation of a module calculated using subsets of the original data. With three LC Stream modules essentially not detectable in the Matadero data, there are clearly differences between populations in the organization of their gene expression networks. However, all modules with a significant association with color (both sexes together) were preserved.

**FIGURE 4:**
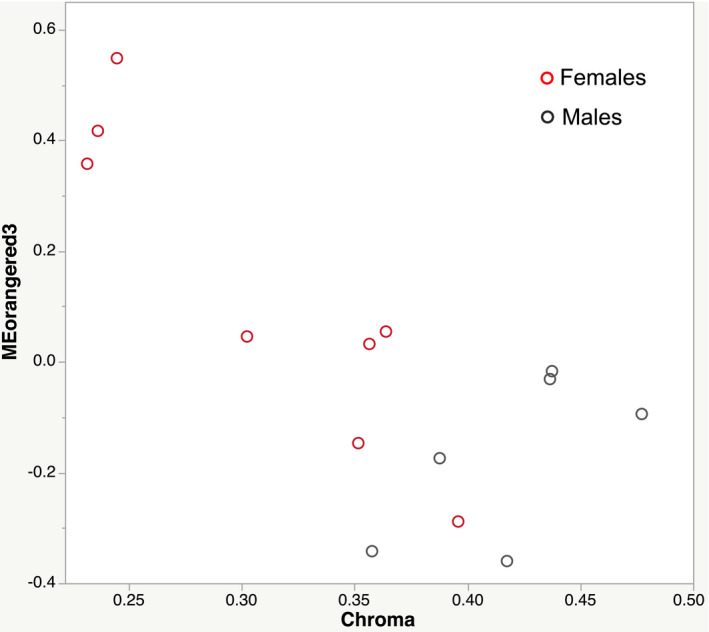
Relationship between red chroma and module eigengene value (first principal component) for “orangered3” in WGCNA analysis of Matadero population, n=14. Females in red, males in dark grey

In the reciprocal analysis of Matadero, 28 modules were generated, of which three showed significant associations with red chroma overall (Table S9). One of these modules was significantly associated with red chroma for females specifically, orangered3 (Figure 4, Table S10; module names are arbitrarily assigned by software). When tested against LC Stream data, Zsummary preservation values ranged from −.36 to 46.1, with four not preserved, three preserved but not strongly, and the remaining 21 strongly preserved. Among the modules not preserved in this analysis was orangered3, the Matadero module most significantly associated with color in females and also significant overall. Thus, the differences between the populations in coexpression patterns were confirmed. The other two color‐associated modules were strongly preserved. Once again all modules exhibited high quality (Table S11).

### 3.4 Do genes showing red chroma‐correlated expression in females show a similar pattern across sexes?

Within population DESeq2 analyses did not provide strong evidence for or against the hypothesis that the same genes mediate variation in red chroma within and between the sexes. When both males and females were included in an analysis of the relationship between red chroma and differential expression for the Matadero population, 275 genes were significant, but these included only 11 of the 28 genes significant in the analysis conducted exclusively with females (Table S12). When a term for sex was added to the analysis along with chroma, just one gene was significant for red chroma, *fgl1*, fibrinogen‐like 1, whereas 375 genes differed in expression between the sexes. Finally, when the interaction between sex and chroma was also included, it was significant for only one gene, *hpdl*, 4‐hydroxyphenylpyruvate dioxygenase‐like (*p*adj = .036).

For the Little Campbell Stream population, when both males and females were included in an analysis of the relationship between red chroma and differential expression, 10 genes were significant (Table S13), though these included only three of the 83 genes significant in the female‐only analysis. Once again, expression was significantly correlated with chroma for fewer genes when a term for sex was added, just five (Table S14), in contrast to 636 that differed between the sexes. When the interaction between sex and chroma was added, it was significant for 13 genes (Table S15), though only two of these were also significant in the analysis confined to females. Thus, there was limited evidence from either population that individual genes showing chroma‐correlated expression in females were similarly correlated with red chroma in males; but there was also little statistically significant evidence that gene expression‐chroma correlations differed between the sexes. Sample sizes likely limited the power of these analyses.

To test whether co‐expressed modules of genes that correlated with chroma in one sex were present in the opposite sex, we conducted WGCNA analyses, focusing on British Columbia stickleback. Analysis of females generated 32 modules, of which eight were significantly correlated with chroma (Table S16). When tested against male data, all modules were preserved and 26 were strongly preserved (Table S17). Analysis of males led to 25 modules, of which two were significantly associated with chroma (Table S18). When tested against female data, all male expression modules were preserved and 20 were strongly preserved (Table S19). Thus, the modules associated with color in each sex were present in the other.

### 3.5 Red females from Little Campbell and Matadero differ from each other and from dull 34 females of other populations in overall and candidate gene expression

**FIGURE 5:**
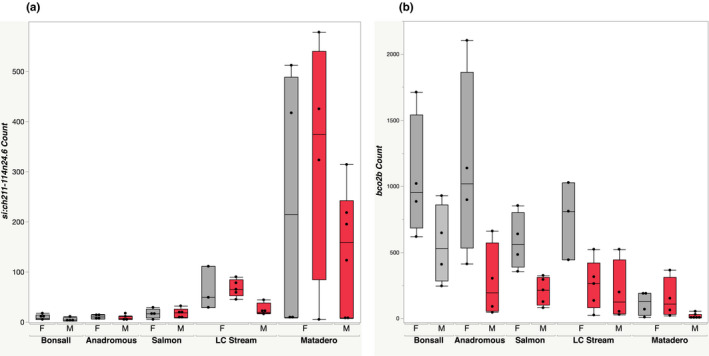
Expression of two genes strongly associated with chroma in females from one or both populations possessing red females, relative to females from populations in which females are mainly dull. Fish classified *a priori* as red in red, dull in gray. Grouped by population, sex (F = female, M = male), and color pattern (red fill indicates colored red in throat, gray indicates dull). Sample size = 51, with 3–6 fish in each sub‐category; (a) *si*:*ch211*‐*114n24*.*6*; (b) *bco2b*

**FIGURE 6:**
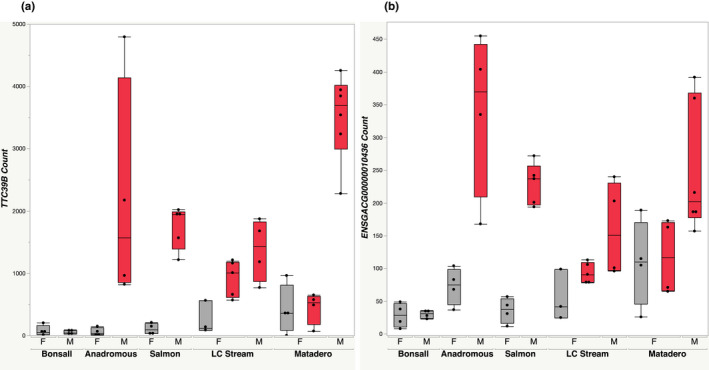
Expression of genes strongly correlated with chroma in inter‐population analyses of males and females. Fish classified *a priori* as red in red, dull in gray. Grouped by population, sex (F = female, M = male). Sample size = 51, with 3–6 fish in each sub‐category. (a) *TTC39B*; (b) ENSGACG00000010436

We evaluated differential expression for each of LC Stream and Matadero red females, relative to dull females from two other populations (Bonsall and Salmon, Table 1; genes identified here were significantly different from females of both dull populations). These patterns of differentiation reflect overall population differences in gene expression, with Matadero fish showing generally greater expression divergence, as expected from the PCA (Figure 2). They also reveal clear population differences in candidate pigmentation gene expression. Differentially expressed candidate genes were largely different for Matadero and LCS females and also from those revealed by within population analyses (Table 1). For Matadero (Table S20), 34 differentially expressed candidate pigmentation genes included *bco2b* (Figure 5), previously implicated in studies of fish carotenoid pigmentation, and *bco1* from the same gene family, as well as *TTC39B* (Figure 6). Just three candidate genes, *TTC39B*, *csf1ra*, and *tmem138*, were differentially expressed by LC Stream red females relative to dull females of other populations (Table S21). *TTC39B*, the only candidate pigmentation gene differentially expressed in both, is located within the credible interval for the Yong et al. (2016) QTL on linkage group IX. ENSGACG00000010436 and *si*:*ch211*‐*114n24*.*6* (Figures 5 and 6), which were both expressed at significantly higher levels in the populations with red females, are not yet well characterized but both are associated with microtubules. The latter is located in a Yong et al. (2016) QTL, on linkage group VI.

**TABLE 1:** Genes differentially expressed for two populations with red females (LCS, Little Campbell Stream; Mat, Matadero), in correlation with red within each population or relative to dull females of two different populations. Numbers of genes from a list of candidate pigmentation genes given first in each cell

	Genes correlated with red within a population: Pigment/All	Genes of red females compared to other population dull females: Pigment/All	Genes correlated with red within a population, and also differentially expressed relative to dull females of other populations: Pigment/All	Genes differentially expressed by red females and also same population males, relative to dull females of two other populations: Pigment/All
LCS Red Females	0/83	3/64	0/0	2/30
Mat Red Females	1/28	34/1283	0/9	29/1049
Both LCS, Mat Red Females	0/0	1/10	0/0	1/7

To more directly evaluate the gene expression differences suggested by these results, we conducted a DESeq2 analysis between Matadero and LC Stream red females. We found that the expression differences were broadly confirmed. Of 1273 genes differentially expressed by Matadero red females when compared with dull females of other populations, but not differentially expressed by LC Stream females in an analogous comparison, 1129 showed significantly different expressions between LC Stream and Matadero red females (Table S20). Of the 54 loci uniquely differentially expressed by LC Stream red females when compared with dull females of other populations, 30 showed significantly different expressions between LC Stream and Matadero red females (Table S21).

For females of the LC Stream population, there was no overlap between the genes showing a correlation between expression and red chroma within LC Stream and those showing differential expression between LC Stream red females and dull females of two other populations (Table 1; Table S21). For Matadero, nine genes from the 28 that were correlated with red within that population were also associated with red between populations. None were on the list of candidate pigmentation genes (Table 1; Table S20).

To gauge the sex specificity of the above results, we also contrasted *males* from LC Stream and Matadero against dull *females* from Bonsall and Salmon populations. Approximately half of the genes differentially expressed in red LC Stream females were also significantly differentially expressed in LC Stream males, relative to dull females. The same was true for 80% of genes for Matadero fish (Table 1, Tables S20 and S21). Matadero males differentially expressed 29 of the same candidate pigmentation genes as females, including some with roles in carotenoid pigmentation (Table S20), notably *bco1*, *bco2b* (Figure 5), and *TTC39B*. For LC Stream, just two candidate pigmentation genes, *TTC39B* and *tmem138* (Crawford et al., 2017) were differentially expressed by both males and red females (Table S21). Thus, *TTC39B* was one of the seven genes, and the only candidate pigmentation gene, differentially expressed by red females and males of both populations relative to dull population females. ENSGACG00000010436, noted above, was also differentially expressed by both sexes and populations (Figure 6).

## 4 DISCUSSION

We analyzed patterns of gene expression in the skin tissue of threespine stickleback from populations in which just males, neither sex, or both sexes may possess red throat coloration. The sexes were distinguished from each other in a principal component analysis of gene expression, and the single California population, the anadromous population, and the British Columbia freshwater populations were also separated. Comparing British Columbia (LC Stream) and California (Matadero) populations possessing convergent female red throat color, we found that different sets of genes were correlated with female chroma in each population, suggesting differences in the underlying mechanisms mediating red intensity. In addition, genes whose expression correlated with female color within populations were mainly different from those associated with color differences between populations. Furthermore, inter‐population contrasts highlighted only a very few shared genes as associated with red female color in both British Columbia and California stickleback.

Previous studies of the genetic basis of stickleback color pattern evolution have generally focused on melanic traits (*e*.*g*. Greenwood et al., 2012; Hart & Miller, 2017), quantitative genetic approaches (*e*.*g*., Bakker et al., 1999), or mendelian analyses based on organismal phenotype (*e*.*g*., Hagen & Moodie, 1979; but see Malek et al., 2012), and males have been studied almost exclusively (but see Yong et al., 2016). To the best of our knowledge, ours is the first study to investigate genomic patterns of expression in stickleback skin toward a better understanding of the genetics of red coloration, and one of the first to use any approach to investigate the contributions of gene expression to variation in female coloration and stickleback dichromatism.

### 4.1 Different genes are associated with red in populations with independently evolved red female coloration

Different genes, associated with different gene ontology terms, were correlated with female chroma in the LC Stream and Matadero populations. But in comparisons of red females with dull females of different populations (Bonsall and Salmon), the pattern was less consistent: distinct genes were implicated for LC Stream and Matadero females in most instances, but not all. At the system level, network analyses confirmed differences in coexpression networks between the Matadero and LC Stream populations, including the module most strongly associated with color in the Matadero fish. This is some of the first evidence suggesting that different genetic mechanisms may contribute to the convergent evolution of female ornaments in different, but closely related, lineages. A difficulty in interpretating these findings, however, is that functional mechanisms for the genes correlated with female chroma within populations remain unknown.

In other studies of stickleback evolving convergently or in parallel as they colonize new habitats, exactly the same mutations have sometimes been recruited from standing genetic variation. Extensive non‐replicated divergence has also been observed, however, including in gene expression data (Bassham et al., 2018; Fang et al., 2020; Jones et al., 2012; Kitano et al., 2019; Roberts‐Kingman et al., 2021).

The principal component analyses suggest substantial differentiation in gene expression of Matadero stickleback compared to the other study populations, a pattern which persisted in almost every analysis. This may be because populations in California have had longer to adapt to freshwater conditions and diverge from other stickleback lineages. California did not suffer the recent glaciation experienced by British Columbia populations and likely represents a separate colonization of freshwater (Colosimo et al., 2005; Jones et al., 2012). Similarly, in a study of gene expression divergence between stream and marine sticklebacks from different continents, more extreme divergence was associated with longer isolation (Kitano et al., 2019). Other potential explanations for the Matadero population's distinctive expression patterns include evolution from a relatively divergent marine ancestor or different selection pressures. Regarding the latter possibility, some differences in environmental selection are to be expected in light of Matadero's location more than 1200 km south of the British Columbia populations.

Somewhat unexpectedly, genes whose expression correlated with red within a population were mainly different from those identified by analogous between population analyses. In particular, clear differences in the expression of carotenoid‐related genes were observed between populations, yet such genes were not associated with variation in red chroma within populations. This result can be explained if some expression differences are necessary for red color in females, but different genes mediate whether, or when, a *potentially* red individual actually expresses such coloration. Such a pattern may be consistent with the plasticity in male nuptial coloration that has been documented in some lake populations in response to fine‐scale variation in the light environment (Brock et al., 2017). At the extreme, plasticity could even explain the differences in which genes were correlated (in expression) with female chroma in the Matadero and LC Stream populations. If different environments during development activated different plastic responses in each population, female chroma could correlate with different sets of genes even without underlying genetic divergence. For example, variation in dietary carotenoids could largely mediate red intensity in one population, whereas parasite loads could be critical in another, each associated with the expression of largely distinct sets of genes. Our inspection of gene lists and ontology terms has not resulted in any straightforward explanations along these lines, although limited understanding of carotenoid pathways and metabolism is constraining.

### 4.2 Variation in expression and coexpression patterns by sex

Our results did not clearly indicate whether the genes showing correlated expression with color in females were associated with color across the sexes, the pattern expected if female coloration evolved as a correlated response to selection on males (Kraaijeveld, 2014; Lande, 1980), rather than independently from males through possibly distinct mechanisms (Lopes‐Ramos et al., 2020; Van der Bijl & Mank, 2021). Within populations, individual genes exhibiting correlated expression with female red coloration showed inconsistent patterns when males and females were analyzed together, yet significant differences between females and males for chroma‐gene expression correlations were also rare. Comparisons between populations with or without red females tended to exhibit more overlap between the sexes in the genes associated with red coloration, but overlap was still far from complete. In network analyses, sets of co‐expressed genes were largely shared between the sexes, including those that correlated with red chroma; but in this analysis and the previous, sample sizes limit our power to identify sex‐specific patterns. Nevertheless, in a few cases, these data provide evidence that specific candidate pigment genes likely influence red chroma in both sexes (discussed below), and patterns at the system level appear to be stronger.

### 4.3 Candidate genes differentiate populations with red females and overlap with known QTL

*TTC39B*was the only candidate pigmentation gene significantly associated with color, in inter‐population analyses, for both sexes of both populations possessing red females. This lipoprotein coding gene is found in a QTL identified by Yong et al. (2016), and thus may also have contributed to QTL results obtained with one of our study populations. Other studies of gene expression in fish suggest a role for *TTC39B* in orange‐red coloration. In the cichlid, *Tropheus duboisi* (Ahi, Lecaudey, Ziegelbecker, Steiner, Glabonjat, et al., 2020), and the clownfish, *Amphiprion ocellaris* (Salis et al., 2019), *TTC39B* has been found to be upregulated in yellow or orange skin, relative to white. In three phylogenetically matched pairs of cichlid species, *TTC39B* was consistently upregulated in the species with red, rather than yellow, skin color (Ahi, Lecaudey, Ziegelbecker, Steiner, Goessler, et al., 2020). Beyond fish, correlations between color and *TTC39B* expression have also been observed in poison frogs (Stuckert et al., 2021). In a hybrid zone of a bird, the long‐tailed finch *Poephila acuticauda*, *TTC39B* cosegregates with carotenoid‐based bill color. This led Hooper et al. (2019; also see Lopez et al., 2021) to hypothesize that, since carotenoids are hydrophobic and require a lipoprotein partnership to be transported to their tissue of deposition, *TTC39B* may play such a role. At a much broader taxonomic scale, a different Tetratricopeptide Repeat gene, *RCP2*, regulates carotenoid accumulation and coloration in monkeyflowers (Stanley et al., 2020). Given the possible contribution of *TTC39B* to red color in Eastern Pacific stickleback populations, its potential role in taxonomically diverse systems, and the limited understanding of the manner in which it might mediate coloration, this gene deserves further investigation.

*si*:*ch211*‐*114n24*.*6* exhibited a striking and unusual expression pattern, being substantially and significantly upregulated in red females (relative to dull fish of other populations) of both populations possessing red females, and to a lesser extent in males and dull females of the same populations. It was minimally expressed in both males and females of the anadromous population, the relatively dull Bonsall population, and the more conventionally dimorphic Salmon River population. *si*:*ch211*‐*114n24*.*6* has close tubulin paralogues such as *tuba8l3*, tubulin, alpha 8 like 3, which is involved in the development of all three pigment cell classes in zebrafish (Patterson & Parichy, 2019). Other tubulin genes may function in pigment granule movement within cells (Ahi, Lecaudey, Ziegelbecker, Steiner, Glabonjat, et al., 2020). Like *TTC39B*, this gene is located in a stickleback color QTL identified by Yong et al. (2016), but on linkage group VI rather than linkage group IX, where *TTC39B* is found. Another gene potentially associated with microtubule organization and a paralogue of *si*:*ch211*‐*114n24*.*6*, ENSGACG00000010436 was also upregulated in red females of both populations (relative to dull females of other populations), though unlike *si*:*ch211*‐*114n24*.*6*, it was expressed at high levels in male anadromous fish, which are red. It has a zebrafish orthologue, *tuba2* (tubulin, alpha 2) that is involved in microtubule organization; it is not located in a Yong et al. (2016) QTL.

Additional genes, previously shown or suggested to affect color patterns, were significantly associated with red female color for just a single population. In particular, *bco2b* was highly significant in the inter‐population analyses for red Matadero females and males. In general, it was expressed at a higher level in the dull fish, as expected for this gene, which breaks down carotenoid pigments. Reduced *bco2b* expression has been linked with persistence and accumulation of carotenoids in studies from chicken through salmon (Ahi, Lecaudey, Ziegelbecker, Steiner, Glabonjat, et al., 2020; Gazda et al., 2020; Lehnert et al., 2019; Toews et al., 2017; Twomey et al., 2020). Over 30 additional candidate pigmentation genes were differentially expressed in red Matadero females relative to dull females of other populations. However, because the Matadero population differed so extensively in expression patterns from the other stream populations, inferring which genes likely play a causal role in coloration is difficult.

In British Columbia's Little Campbell population, *csf1ra* was significantly elevated in red‐throated females, relative to dull females of other populations, but it was not similarly elevated in red Matadero females (although it was in Matadero males). This gene influences carotenoid‐based coloration through roles in the development of xanthophores/erythrophores (Kottler et al., 2013; Patterson & Parichy, 2019; Salzburger et al., 2007).

### 4.4 Tissue‐specific and developmental timing of expression of key genes—limitations of current data

A caveat regarding our data is that they are from a single tissue at a single developmental point. Important processes in color pattern development may well take place prior to the stage examined here. Similarly, in the liver and in other organs and tissues, the expression of key genes may be different from in the skin, and play a role in mediating the relative abundance of the pigments responsible for color patterns (Patterson & Parichy, 2019; but see Gazda et al., 2020). In addition, we did not attempt to evaluate alternative splicing (e.g., Howes et al., 2017).

## 5 CONCLUSIONS

We find that the genes showing correlated expression with female red coloration in stickleback differ extensively between populations. Our results also suggest that the genes mediating variation in female red coloration within populations are largely different from those associated with differences between populations. However, it was unclear from our data whether genes associated with female red chroma were similarly correlated across the sexes, or differed between males and females. Several candidate pigmentation genes from studies of other taxa were identified in our inter‐population analyses but very few from analyses within populations. *TTC39B*, located in a QTL for female red coloration for one of our study populations, showed the most consistent patterns. It will be important to build on our investigation of naturally occurring variation in gene expression with manipulations using CRISPR and related methods, especially for *TTC39B*.

## CONFLICT OF INTEREST

The authors declare no conflict of interest.

## AUTHOR CONTRIBUTIONS

**Jeff S. McKinnon:** Conceptualization (equal); Data curation (supporting); Formal analysis (equal); Funding acquisition (equal); Investigation (supporting); Methodology (equal); Project administration (lead); Resources (equal); Supervision (equal); Writing – original draft (lead). **William Burns Newsome:** Conceptualization (supporting); Data curation (equal); Formal analysis (equal); Investigation (lead); Methodology (supporting); Writing – original draft (equal). **Christopher N. Balakrishnan:** Conceptualization (equal); Data curation (lead); Formal analysis (equal); Funding acquisition (equal); Investigation (supporting); Methodology (lead); Project administration (equal); Resources (equal); Supervision (equal); Writing – review & editing (equal).

## Supporting information

Table S1

Table S2

Table S3

Table S4

Table S5

Table S6

Table S7

Table S8

Table S9

Table S10

Table S11

Table S12

Table S13

Table S14

Table S15

Table S16

Table S17

Table S18

Table S19

Table S20

Table S21

## Data Availability

Expression data are available at https://www.ncbi.nlm.nih.gov/bioproject/PRJNA646862. Additional data that support the findings of this study have been deposited in Dryad: https://doi.org/10.5061/dryad.sn02v6x64.

## References

[ece38860-bib-0001] Ahi, E. P. , Lecaudey, L. A. , Ziegelbecker, A. , Steiner, O. , Glabonjat, R. , Goessler, W. , Hois, V. , Wagner, C. , Lass, A. , & Sefc, K. M. (2020). Comparative transcriptomics reveals candidate carotenoid color genes in an East African cichlid fish. BMC Genomics, 21, 54. 10.1186/s12864-020-6473-8 31948394 PMC6966818

[ece38860-bib-0002] Ahi, E. P. , Lecaudey, L. A. , Ziegelbecker, A. , Steiner, O. , Goessler, R. , & Sefc, K. F. (2020). Expression levels of the tetratricopeptide repeat protein gene *ttc39b* covary with carotenoid‐based skin colour in cichlid fish. Biology Letters, 16, 20200629. 33236977 10.1098/rsbl.2020.0629 PMC7728679

[ece38860-bib-0003] Anders, S. , & Huber, W. (2010). Differential expression analysis for sequence count data. Genome Biology, 11, R106. 10.1186/gb-2010-11-10-r106 20979621 PMC3218662

[ece38860-bib-0004] Anders, S. , Pyl, P. T. , & Huber, W. (2015). HTSeq‐A Python framework to work with high‐throughput sequencing data. Bioinformatics, 31, 166–169. 10.1093/bioinformatics/btu638 25260700 PMC4287950

[ece38860-bib-0005] Andersson, M. (1994). Sexual selection. Princeton University Press.

[ece38860-bib-0006] Badyaev, A. V. , & Hill, G. E. (2003). Avian sexual dichromatism in relation to phylogeny and ecology. Annual Review of Ecology, Evolution, and Systematics, 34, 27–49. 10.1146/annurev.ecolsys.34.011802.132441

[ece38860-bib-0007] Bakker, T. C. M. (1993). Positive genetic correlation between female preference and preferred male ornament in sticklebacks. Nature, 363, 255–257. 10.1038/363255a0

[ece38860-bib-0008] Bakker, T. C. M. (1994). Evolution of aggressive behaviour in the threespine stickleback. In M. A. Bell , & S. A. Foster (Eds.), The evolutionary biology of the threespine stickleback (pp. 345–380). Oxford University Press.

[ece38860-bib-0009] Bakker, T. C. M. , Künzler, R. , & Mazzi, D. (1999). Condition‐related mate choice in sticklebacks. Nature, 401, 234. 10.1038/45727

[ece38860-bib-0010] Bassham, S. , Catchen, J. , Lescak, E. , von Hippel, F. A. , & Cresko, W. A. (2018). Repeated selection of alternatively adapted haplotypes creates sweeping genomic remodeling in Stickleback. Genetics, 209, 921–939. 10.1534/genetics.117.300610 29794240 PMC6028257

[ece38860-bib-0011] Baxter, L. L. , Watkins‐Chow, D. E. , Pavan, W. J. , & Loftus, S. K. (2019). A curated gene list for expanding the horizons of pigmentation biology. Pigment Cell & Melanoma Research, 32, 348–358. 10.1111/pcmr.12743 30339321 PMC10413850

[ece38860-bib-0012] Benjamini, Y. , & Hochberg, Y. (1995). Controlling the false discovery rate: A practical and powerful approach to multiple hypothesis testing. Journal of the Royal Statistical Society: Series B, 57, 289–300.

[ece38860-bib-0013] Blouw, D. M. , & Hagen, D. (1990). Breeding ecology and evidence of reproductive isolation of a widespread stickleback fish (Gasterosteidae) in Nova Scotia, Canada. Biological Journal of the Linnean Society, 39, 195–217. 10.1111/j.1095-8312.1990.tb00512.x

[ece38860-bib-0014] Boughman, J. W. (2001). Divergent sexual selection enhances reproductive isolation in sticklebacks. Nature, 411, 944–948. 11418857 10.1038/35082064

[ece38860-bib-0015] Brock, C. , Cummings, M. , & Bolnick, D. (2017). Phenotypic plasticity drives a depth gradient in male conspicuousness in threespine stickleback, *Gasterosteus aculeatus* . Evolution, 71, 2022–2036. 10.1111/evo.13282 28590028

[ece38860-bib-0016] Brock, C. D. , Rennison, D. , Veen, T. , & Bolnick, D. I. (2018). Opsin expression predicts male nuptial color in threespine stickleback. Ecology and Evolution, 8, 7094–7102. 10.1002/ece3.4231 30073070 PMC6065272

[ece38860-bib-0017] Candolin, U. , & Tukiainen, I. (2015). The sexual selection paradigm: have we overlooked other mechanisms in the evolution of male ornaments? Proceedings of the Royal Society B: Biological Sciences, 282, 20151987. 10.1098/rspb.2015.1987 PMC4614788 26446811

[ece38860-bib-0018] Colosimo, P. F. , Hosemann, K. E. , Balabhadra, S. , Villarreal, G. , Dickson, M. , Grimwood, J. , Schmutz, J. , Myers, R. M. , Schluter, D. , & Kingsley, D. M. (2005). Widespread parallel evolution in sticklebacks by repeated fixation of ectodysplasin alleles. Science, 307, 1928–1933. 10.1126/science.1107239 15790847

[ece38860-bib-0019] Crawford, N. G. , Kelly, D. E. , Hansen, M. E. B. , Beltrame, M. H. , Fan, S. , Bowman, S. L. , Jewett, E. , Ranciaro, A. , Thompson, S. , Lo, Y. , Pfeifer, S. P. , Jensen, J. D. , Campbell, M. C. , Beggs, W. , Hormozdiari, F. , Mpoloka, S. W. , Mokone, G. G. , Nyambo, T. , Meskel, D. W. , … Tishkoff, S. A. (2017). Loci associated with skin pigmentation identified in African populations. Science, 358(eaan8433), 10.1126/science.aan8433 PMC5759959 29025994

[ece38860-bib-0020] Cuthill, I. C. , Allen, W. L. , Arbuckle, K. , Caspers, B. , Chaplin, G. , Hauber, M. E. , Hill, G. E. , Jablonski, N. G. , Jiggins, C. D. , Kelber, A. , Mappes, J. , Marshall, J. , Merrill, R. , Osorio, D. , Prum, R. , Roberts, N. W. , Roulin, A. , Rowland, H. M. , Sherratt, T. N. , … Caro, T. (2017). The biology of color. Science, 357. 10.1126/science.aan0221 28774901

[ece38860-bib-0021] Darwin, C. (1871). The descent of man, and selection in relation to sex. John Murray.

[ece38860-bib-0022] Eden, E. , Lipson, D. , Yogev, S. , & Yakhini, Z. (2007). Discovering motifs in ranked lists of DNA sequences. PLoS Computational Biology, 3, e39. 10.1371/journal.pcbi.0030039 17381235 PMC1829477

[ece38860-bib-0023] Eden, E. , Navon, R. , Steinfeld, I. , Lipson, D. , & Yakhini, Z. (2009). GOrilla: A tool for discovery and visualization of enriched GO terms in ranked gene lists. BMC Bioinformatics, 10, 48. 10.1186/1471-2105-10-48 19192299 PMC2644678

[ece38860-bib-0024] Fang, B. , Kemppainen, P. , Momigliano, P. , Feng, X. , & Merilä, J. (2020). On the causes of geographically heterogeneous parallel evolution in sticklebacks. Nature Ecology and Evolution, 4, 1105–1115. 10.1038/s41559-020-1222-6 32572218

[ece38860-bib-0025] Gazda, M. A. , Araújo, P. M. , Lopes, R. J. , Toomey, M. B. , Andrade, P. , Afonso, S. , Marques, C. , Nunes, L. , Pereira, P. , Trigo, S. , Hill, G. E. , Corbo, J. C. , & Carneiro, M. (2020). A genetic mechanism for sexual dichromatism in birds. Science, 368, 1270–1274. 10.1126/science.aba0803 32527835

[ece38860-bib-0026] Glazer, A. M. , Killingbeck, E. E. , Mitros, T. , Rokhsar, D. S. , & Miller, C. T. (2015). Genome assembly improvement and mapping convergently evolved skeletal traits in sticklebacks with genotyping‐by‐sequencing. Genes, Genomes, Genetics, 5, 1463–1472. 26044731 10.1534/g3.115.017905 PMC4502380

[ece38860-bib-0027] Greenwood, A. K. , Cech, J. N. , & Peichel, C. L. (2012). Molecular and developmental contributions to divergent pigment patterns in marine and freshwater sticklebacks. Evolution & Development, 14, 351–362. 10.1111/j.1525-142X.2012.00553.x 22765206 PMC3394544

[ece38860-bib-0028] Greenwood, A. K. , Jones, F. C. , Chan, Y. F. , Brady, S. D. , Absher, D. M. , Grimwood, J. , Schmutz, J. , Myers, R. M. , Kingsley, D. M. , & Peichel, C. L. (2011). The genetic basis of divergent pigment patterns in juvenile threespine sticklebacks. Heredity, 107, 155–166. 10.1038/hdy.2011.1 21304547 PMC3136628

[ece38860-bib-0029] Gygax, M. , Rentsch, A. K. , Rudman, S. M. , & Rennison, D. J. (2018). Differential predation alters pigmentation in threespine stickleback (*Gasterosteus aculeatus*). Journal of Evolutionary Biology, 31, 1589–1598. 10.1111/jeb.13354 30055069

[ece38860-bib-0030] Hagen, D. W. , & Moodie, G. (1979). Polymorphism for breeding colors in *Gasterosteus aculeatus*. 1, their genetics and geographic distribution. Evolution, 33, 641–648. 10.2307/2407787 28563943

[ece38860-bib-0031] Hare, L. M. , & Simmons, L. W. (2019). Sexual selection and its evolutionary consequences in female animals. Biological Reviews, 94, 929–956. 10.1111/brv.12484 30484943

[ece38860-bib-0032] Hart, J. C. , & Miller, C. T. (2017). Sequence‐based mapping and genome editing reveal mutations in stickleback *Hps5* cause oculocutaneous albinism and the casper Phenotype. Genes, Genomes, Genetics, 7, 3123–3131. 28739598 10.1534/g3.117.1125 PMC5592937

[ece38860-bib-0033] Hernández, A. , Martínez‐Gómez, M. , Beamonte‐Barrientos, R. , & Montoya, B. (2021). Colourful traits in female birds relate to individual condition, reproductive performance and male‐mate preferences: a meta‐analytic approach. Biology Letters, 17, 20210283. 10.1098/rsbl.2021.0283 34493064 PMC8424322

[ece38860-bib-0034] Hill, G. E. (1991). Plumage coloration is a sexually selected indicator of male quality. Nature, 350, 337–339. 10.1038/350337a0

[ece38860-bib-0035] Hooper, D. M. , Griffith, S. C. , & Price, T. D. (2019). Sex chromosome inversions enforce reproductive isolation across an avian hybrid zone. Molecular Ecology, 28, 1246–1262. 10.1111/mec.14874 30230092

[ece38860-bib-0036] Howes, T. R. , Summers, B. R. , & Kingsley, D. M. (2017). Dorsal spine evolution in threespine sticklebacks via a splicing change in MSX2A. BMC Biology, 15, 115. 10.1186/s12915-017-0456-5 29212540 PMC5719529

[ece38860-bib-0037] Johnson, S. , & Candolin, U. (2017). Predation cost of a sexual signal in the threespine stickleback. Behavioral Ecology, 28, 1160–1165. 10.1093/beheco/arx080

[ece38860-bib-0038] Jones, F. C. , Grabherr, M. G. , Chan, Y. F. , Russell, P. , Mauceli, E. , Johnson, J. , Swofford, R. , Pirun, M. , Zody, M. C. , White, S. , Birney, E. , Searle, S. , Schmutz, J. , Grimwood, J. , Dickson, M. C. , Myers, R. M. , Miller, C. T. , Summers, B. R. , Knecht, A. K. , … Kingsley, D. M. (2012). The genomic basis of adaptive evolution in threespine sticklebacks. Nature, 484, 55–61. 10.1038/nature10944 22481358 PMC3322419

[ece38860-bib-0039] Kettlewell, H. B. D. (1955). Selection experiments on industrial melanism in the Lepidoptera. Heredity, 9, 323–342. 10.1038/hdy.1955.36

[ece38860-bib-0040] Kitano, J. , Ishikawa, A. , & Kusakabe, M. (2019). Parallel transcriptome evolution in stream threespine sticklebacks. Development, Growth & Differentiation, 61, 104–113. 10.1111/dgd.12576 30393863

[ece38860-bib-0041] Koch, R. E. , & Hill, G. E. (2018). Do carotenoid‐based ornaments entail resource trade‐offs? An evaluation of theory and data. Functional Ecology, 32, 1908–1920. 10.1111/1365-2435.13122

[ece38860-bib-0042] Kottler, V. A. , Fadeev, A. , Weigel, D. , & Dreyer, C. (2013). Pigment pattern formation in the guppy, *Poecilia reticulata*, involves the Kita and Csf1ra receptor tyrosine kinases. Genetics, 194, 631–646. 10.1534/genetics.113.151738 23666934 PMC3697969

[ece38860-bib-0043] Kraaijeveld, K. (2014). Reversible trait loss: the genetic architecture of female ornaments. Annual Review of Ecology, Evolution, and Systematics, 45, 159–177. 10.1146/annurev-ecolsys-120213-091550

[ece38860-bib-0044] Krueger, F. (2015). Trim Galore. A wrapper tool around Cutadapt and FastQC to consistently apply quality and adapter trimming to FastQ files. Babraham Institute.

[ece38860-bib-0045] Lande, R. (1980). Sexual dimorphism, sexual selection, and adaptation in polygenic characters. Evolution, 34, 292–305. 10.1111/j.1558-5646.1980.tb04817.x 28563426

[ece38860-bib-0046] Langfelder, P. , & Horvath, S. (2008). WGCNA: an R package for weighted correlation network analysis. BMC Bioinformatics, 9, 559. 10.1186/1471-2105-9-559 19114008 PMC2631488

[ece38860-bib-0047] Langfelder, P. , Luo, R. , Oldham, M. C. , & Horvath, S. (2011). Is my network module preserved and reproducible? PLoS Computational Biology, 7, e1001057. 10.1371/journal.pcbi.1001057 21283776 PMC3024255

[ece38860-bib-0048] Lehnert, S. J. , Christensen, K. A. , Vandersteen, W. E. , Sakhrani, D. , Pitcher, T. E. , Heath, J. W. , Koop, B. F. , Heath, D. D. , & Devlin, R. H. (2019). Carotenoid pigmentation in salmon: variation in expression at BCO2‐l locus controls a key fitness trait affecting red coloration. Proceedings of the Royal Society B, 286, 20191588. 10.1098/rspb.2019.1588 31615356 PMC6834058

[ece38860-bib-0049] Lopes‐Ramos, C. M. , Chen, C.‐Y. , Kuijjer, M. L. , Paulson, J. N. , Sonawane, A. R. , Fagny, M. , Platig, J. , Glass, K. , Quackenbush, J. , & DeMeo, D. L. (2020). Sex differences in gene expression and regulatory networks across 29 human tissues. Cell Reports, 31, 107795. 10.1016/j.celrep.2020.107795 32579922 PMC7898458

[ece38860-bib-0050] Lopez, K. A. , McDiarmid, C. S. , Griffith, S. C. , Lovette, I. J. , & Hooper, D. M. (2021). Evaluating evidence of mitonuclear incompatibilities with the sex chromosomes in an avian hybrid zone. Evolution, 75, 1395–1414. 10.1111/evo.14243 33908624

[ece38860-bib-0051] Maia, R. , Eliason, C. M. , Bitton, P. P. , Doucet, S. M. , & Shawkey, M. D. (2013). pavo: An R package for the analysis, visualization and organization of spectral data. Methods in Ecology and Evolution, 4, 906–913.

[ece38860-bib-0052] Malek, T. , Boughman, J. W. , Dworkin, I. , & Peichel, C. L. (2012). Admixture mapping of male nuptial colour and body shape in a recently formed hybrid population of threespine stickleback. Molecular Ecology, 21, 5265–5279. 10.1111/j.1365-294X.2012.05660.x 22681397 PMC3443501

[ece38860-bib-0053] Marques, D. A. , Lucek, K. , Haesler, M. P. , Feller, A. F. , Meier, J. I. , Wagner, C. E. , Excoffier, L. , & Seehausen, O. (2017). Genomic landscape of early ecological speciation initiated by selection on nuptial colour. Molecular Ecology, 26, 7–24. 27483035 10.1111/mec.13774

[ece38860-bib-0054] Martin, M. (2011). Cutadapt removes adapter sequences from high‐throughput sequencing reads. EMBnet.Journal, 17, 10. 10.14806/ej.17.1.200

[ece38860-bib-0055] McKinnon, J. S. , deMayo, R. F. , Granquist, R. , & Weggel, L. (2000). Female red throat coloration in two populations of threespine stickleback. Behaviour, 137, 947–963. 10.1163/156853900502556

[ece38860-bib-0056] McKinnon, J. S. , Kitano, J. , & Aubin‐Horth, N. (2019). *Gasterosteus*, *Anolis*, *Mus* and more: the changing roles of vertebrate models in evolution and behavior. Evolutionary Ecology Research, 20, 1–25.

[ece38860-bib-0057] McKinnon, J. S. , & Pierotti, M. (2010). Color polymorphism and correlated characters: genetic mechanisms and evolution. Molecular Ecology, 19, 5101–5125. 21040047 10.1111/j.1365-294X.2010.04846.x

[ece38860-bib-0058] McKinnon, J. S. , & Rundle, H. D. (2002). Speciation in nature: the threespine stickleback model systems. Trends in Ecology & Evolution, 17, 480–488. 10.1016/S0169-5347(02)02579-X

[ece38860-bib-0059] McLean, C. A. , Lutz, A. , Rankin, K. J. , Stuart‐Fox, D. , & Moussalli, A. (2017). Revealing the biochemical and genetic basis of color variation in a polymorphic lizard. Molecular Biology and Evolution, 34, 1924–1935. 10.1093/molbev/msx136 28431132

[ece38860-bib-0060] Milinski, M. , & Bakker, T. C. (1990). Female sticklebacks use male coloration in mate choice and hence avoid parasitized males. Nature, 344, 330–333. 10.1038/344330a0

[ece38860-bib-0061] Miller, C. T. , Beleza, S. , Pollen, A. A. , Schluter, D. , Kittles, R. A. , Shriver, M. D. , & Kingsley, D. M. (2007). cis‐Regulatory changes in Kit Ligand expression and parallel evolution of pigmentation in sticklebacks and humans. Cell, 131, 1179–1189. 10.1016/j.cell.2007.10.055 18083106 PMC2900316

[ece38860-bib-0062] Olendorf, R. , Rodd, F. H. , Punzalan, D. , Houde, A. E. , Hurt, C. , Reznick, D. N. , & Hughes, K. A. (2006). Frequency‐dependent survival in natural guppy populations. Nature, 441, 633–636. 10.1038/nature04646 16738659

[ece38860-bib-0063] Orteau, A. , & Jiggins, C. D. (2020). The genomics of coloration provides insights into adaptive evolution. Nature Reviews Genetics, 21(8), 461–475. 10.1038/s41576-020-0234-z 32382123

[ece38860-bib-0064] Patterson, L. B. , & Parichy, D. M. (2019). Zebrafish pigment pattern formation: insights into the development and evolution of adult form. Annual Review of Genetics, 2019(53), 505–530. 10.1146/annurev-genet-112618-043741 31509458

[ece38860-bib-0065] R Core Team (2017). R: A language and environment for statistical computing. R Foundation for Statistical Computing. https://www.R‐project.org/

[ece38860-bib-0066] Reid, K. , Bell, M. A. , & Veeramah, K. R. (2021). Threespine stickleback: a model system for evolutionary genomics. Annual Review of Genomics and Human Genetics, 22(1), 357–383. 10.1146/annurev-genom-111720-081402 PMC8415275 33909459

[ece38860-bib-0067] Reimchen, T. E. (1989). Loss of nuptial color in threespine sticklebacks (*Gasterosteus aculeatus*). Evolution, 43, 450–460. 28568546 10.1111/j.1558-5646.1989.tb04239.x

[ece38860-bib-0068] Roberts Kingman, G. A. , Vyas, D. N. , Jones, F. C. , Brady, S. D. , Chen, H. I. , Reid, K. , Milhaven, M. , Bertino, T. S. , Aguirre, W. E. , Heins, D. C. , von Hippel, F. A. , Park, P. J. , Kirch, M. , Absher, D. M. , Myers, R. M. , Di Palma, F. , Bell, M. A. , Kingsley, D. M. , & Veeramah, K. R. (2021). Predicting future from past: The genomic basis of recurrent and rapid stickleback evolution. Science Advances, 7(25). 10.1126/sciadv.abg5285 PMC8213234 34144992

[ece38860-bib-0069] Rush, V. N. , McKinnon, J. S. , Abney, M. A. A. , & Sargent, R. C. (2003). Reflectance spectra from free‐swimming sticklebacks (*Gasterosteus*): social context and eye‐jaw contrast. Behaviour, 140, 1003–1019. 10.1163/156853903322589614

[ece38860-bib-0070] Salis, P. , Lorin, T. , Lewis, V. , Rey, C. , Marcionetti, A. , Escande, M.‐L. , Roux, N. , Besseau, L. , Salamin, N. , Sémon, M. , Parichy, D. , Volff, J.‐N. , & Laudet, V. (2019). Developmental and comparative transcriptomic identification of iridophore contribution to white barring in clownfish. Pigment Cell & Melanoma Research, 2019(32), 391–402. 10.1111/pcmr.12766 PMC6483885 30633441

[ece38860-bib-0071] Salzburger, W. , Braasch, I. , & Meyer, A. (2007). Adaptive sequence evolution in a color gene involved in the formation of the characteristic egg‐dummies of male haplochromine cichlid fishes. BMC Biology, 5, 1. 10.1186/1741-7007-5-51 18005399 PMC2254590

[ece38860-bib-0072] Stanley, L. E. , Ding, B. , Su, W. , Mou, F. , Hill, C. , Chen, S. , & Yuan, Y.‐W. (2020). A Tetratricopeptide repeat protein regulates carotenoid biosynthesis and chromoplast development in monkeyflowers (*Mimulus*). The Plant Cell, 32, 1536–1555. 32132132 10.1105/tpc.19.00755 PMC7203930

[ece38860-bib-0073] Stuckert, A. M. M. , Chouteau, M. , McClure, M. , LaPolice, T. M. , Linderoth, T. , Nielsen, R. , Summers, K. , & MacManes, M. D. (2021). The genomics of mimicry: gene expression throughout development provides insights into convergent and divergent phenotypes in a Müllerian mimicry system. Molecular Ecology, 30, 4039–4061. 10.1111/mec.16024 34145931 PMC8457190

[ece38860-bib-0074] Stuckert, A. M. M. , Drury, S. , Anderson, C. M. , Bowling, T. B. T. , & McKinnon, J. S. (2019). Evolution and assessment of colour patterns in stream‐resident and anadromous male threespine stickleback from three regions. Journal of Fish Biology, 94, 520–525. 30693501 10.1111/jfb.13913

[ece38860-bib-0075] Stuckert, A. M. M. , Moore, E. , Coyle, K. P. , Davison, I. , MacManes, M. D. , Roberts, R. , & Summers, K. (2019). Variation in pigmentation gene expression is associated with distinct aposematic color morphs in the poison frog *Dendrobates auratus* . BMC Evolutionary Biology, 19, 85. 10.1186/s12862-019-1410-7 30995908 PMC6472079

[ece38860-bib-0076] Tinghitella, R. M. , Lehto, W. R. , & Lierheimer, V. F. (2018). Color and behavior differently predict competitive outcomes for divergent stickleback color morphs. Current Zoology, 64, 115–123. 10.1093/cz/zox070 29492044 PMC5809037

[ece38860-bib-0077] Toews, D. P. L. , Hofmeister, N. R. , & Taylor, S. A. (2017). The evolution and genetics of carotenoid processing in animals. Trends in Genetics, 33(3), 171–182. 10.1016/j.tig.2017.01.002 28174022

[ece38860-bib-0078] Trapnell, C. , Pachter, L. , & Salzberg, S. L. (2009). TopHat: discovering splice junctions with RNA‐Seq. Bioinformatics, 25, 1105–1111. 10.1093/bioinformatics/btp120 19289445 PMC2672628

[ece38860-bib-0079] Twomey, E. , Johnson, J. D. , Castroviejo‐Fisher, S. , & Van Bocxlaer, I. (2020). A ketocarotenoid‐based colour polymorphism in the Sira poison frog *Ranitomeya sirensis* indicates novel gene interactions underlying aposematic signal variation. Molecular Ecology, 29, 2004–2015. 10.1111/mec.15466 32402099

[ece38860-bib-0080] Van der Bijl, W. , & Mank, J. E. (2021). Widespread cryptic variation in genetic architecture between the sexes. Evolution Letters, 5(4), 359–369. 10.1002/evl3.245 34367661 PMC8327960

[ece38860-bib-0081] Von Hippel, F. A. (1999). Black male bellies and red female throats: color changes with breeding status in a threespine stickleback. Environmental Biology of Fishes, 55, 237–244. 10.1023/A:1007572620424

[ece38860-bib-0082] Wright, D. S. , Pierotti, M. E. , Rundle, H. , & McKinnon, J. S. (2015). Conspicuous female ornamentation and male mate preference in threespine sticklebacks (*Gasterosteus aculeatus*). PLoS One, 10(3), e0120723. 25806520 10.1371/journal.pone.0120723 PMC4373685

[ece38860-bib-0083] Wright, D. S. , Yong, L. , Pierotti, M. E. , & McKinnon, J. S. (2016). Relationships between male red throat coloration, pelvic spine coloration, and courtship behaviors in threespine stickleback. Evolutionary Ecology Research, 17, 407–418.

[ece38860-bib-0084] Yassin, A. , Delaney, E. K. , Reddiex, A. J. , Seher, T. D. , Bastide, H. , Appleton, N. C. , Lack, J. B. , David, J. R. , Chenoweth, S. F. , Pool, J. E. , & Kopp, A. (2016). The pdm3 Locus is a hotspot for recurrent evolution of female‐limited color dimorphism in *Drosophila* . Current Biology, 26, 2412–2422. 10.1016/j.cub.2016.07.016 27546577 PMC5450831

[ece38860-bib-0085] Yong, L. , Guo, R. , Wright, D. S. , Mears, S. A. , Pierotti, M. , & McKinnon, J. S. (2013). Correlates of red throat coloration in female stickleback and their potential evolutionary significance. Evolutionary Ecology Research, 15, 453–472.

[ece38860-bib-0086] Yong, L. , Lee, B. , & McKinnon, J. S. (2018). Variation in female aggression in 2 three‐spined stickleback populations with female throat and spine coloration. Current Zoology, 2018, 1–6. 10.1093/cz/zoy020 PMC6007237 30402077

[ece38860-bib-0087] Yong, L. , Peichel, C. L. , & McKinnon, J. S. (2016). Genetic architecture of conspicuous red ornaments in female threespine stickleback. Genes, Genomes, Genetics, 6, 579–588. 10.1534/g3.115.024505 PMC4777121 26715094

[ece38860-bib-0088] Yong, L. , Woodall, B. , Pierotti, M. E. R. , & McKinnon, J. S. (2015). Intrasexual competition and ornament evolution in female threespine sticklebacks. Behavioral Ecology, 26, 1030–1038.

